# Telehealth Curricula in the Pediatric Core Clerkship: Results From a Survey of Clerkship Directors

**DOI:** 10.7759/cureus.39200

**Published:** 2023-05-18

**Authors:** Tina Kumra, Danielle B Amundsen, Alexa Mullins, Daniel J Hindman, Helen K Hughes, Amit K Pahwa

**Affiliations:** 1 Department of Pediatrics, Johns Hopkins University School of Medicine, Baltimore, USA

**Keywords:** curriculum development and evaluation, pediatrics medicine, telemedicine (tm), telehealth education, undergraduate and graduate medical education

## Abstract

Objective: Given the increasing prevalence of telehealth, medical students require dedicated instruction in the practice of high-quality telehealth. This study characterizes telehealth practices and curricula in pediatric core clerkships across the United States and Canada.

Methods: We surveyed pediatric core clerkship directors and site directors through the 2020 Council on Medical Student Education in Pediatrics (COMSEP) annual member survey. We analyzed the results using descriptive statistics.

Results: Of 104 medical schools represented, 28 responded (26.9%). Directors reported students spent little time on telehealth during their pediatric core clerkships (average 8.2% of clerkship; SD 10.4). Only 10.7% (n=3) of clerkships had dedicated telehealth curricula. The instructional methods, content, and modes of evaluation varied across the clerkships’ curricula. Barriers to implementation of telehealth curricula included lack of dedicated time in the existing curriculum (64.0%), lack of faculty time to teach (44.0%), lack of curricular materials (44.0%), students not participating in telehealth activities (40.0%) and lack of faculty expertise (36.0%).

Conclusions: Most pediatric core clerkships do not include dedicated telehealth curricula, and the characteristics of existing curricula vary. Considering the rapid adoption of telemedicine, pediatric core clerkships merit additional support and guidance for the training of medical students in telehealth practice.

## Introduction

Even before the coronavirus disease 2019 (COVID-19) pandemic, many medical societies began calling for telehealth education. In 2016, the American Medical Association (AMA) released a policy encouraging the incorporation of telemedicine competencies in both undergraduate and graduate medical education [1]. Similarly, in 2015, the American Academy of Pediatrics (AAP) advocated increasing the appropriate use of telehealth and, in doing so, improve access to care for medically underserved populations [2]. However, only 56.3% of medical schools reported having telehealth curricula in 2019 [3]. Prior to the COVID-19 pandemic, the bulk of telehealth curricula and telemedicine experiences occurred during clerkships [4]. Telehealth curricula focused most on electronic patient communication, such as use of patient portals, while only half included teaching on synchronous video visits [3].

Changes in state and federal policy and insurance coverage to adapt to the COVID-19 pandemic exponentially increased the use of telehealth [5-7]. Thus, medical students increasingly participated in telemedicine, necessitating training in the appropriate performance of telephone and video visits [8]. Simultaneously, the American Association of Medical Colleges (AAMC) developed telemedicine competencies [9]. However, it is unclear if and how schools adapted curricula to respond to these developments.

These problems are not unique to pediatrics. A recent survey of internal medicine clerkship directors demonstrated that, before the pandemic, no schools offered telehealth curricula in the internal medicine core clerkship [10]. However, by the early pandemic, 40% of schools offered telehealth instruction due to clinical interruption which then fell to 24.7% of schools as of October 2020 [10]. In this study, we present the findings of a cross-sectional survey of pediatric core clerkship directors regarding the use of telehealth and the prevalence of telehealth curricula during medical students’ core clerkships in pediatrics.

## Materials and methods

Each spring, the Council on Medical Student Education in Pediatrics (COMSEP) surveys its members on several topics related to undergraduate medical education (UME). COMSEP is an international professional society of educators focused on UME in pediatrics [11]. The organization invites members to submit questions to be included in the annual survey.

Using standard principles of survey design, we developed a brief de novo survey instrument focused on telehealth use and instruction during the pediatric core clerkship. We reviewed the survey with the directors of telemedicine at Johns Hopkins Health System for content and conducted cognitive interviews with three internal medicine clerkship directors to assess clarity. An additional round of feedback was performed with the COMSEP survey committee, which approved six questions for final distribution in a larger survey to all members. The questions were administered only to those respondents who identified as a pediatric core clerkship director or a pediatric core clerkship site director. Schools with responses from multiple clerkship directors were excluded. When both a clerkship director and one or more site directors responded, only the clerkship director’s responses were included. Using COMSEP membership data, we estimated the study population to be 107 medical schools represented by 107 core clerkship directors. We were unable to confirm the number of additional schools represented by only site director members. Questions focused on the portion of clinical time medical students spend delivering care via telemedicine, the presence of instruction in telehealth, methods and content of instruction, assessment of telehealth skills, and barriers to implementing a telehealth curriculum.

The survey was distributed through LimeSurvey (Hamburg, Germany). COMSEP members were electronically invited to complete the survey, and those that initially did not respond were sent up to two reminders. The survey opened on April 14, 2021, and closed on May 14, 2021. No incentives were offered for participation. COMSEP provided the study team with deidentified responses. We used STATA (College Station, TX, USA) to analyze the data and perform descriptive statistics. This study was approved as an exemption after formal review by the Johns Hopkins Medicine Institutional Review Board (#00279811).

## Results

The complete survey was distributed to 104 pediatric core clerkship directors. The response rate for the telehealth questions was 26.9% (28/104). Complete demographics are available in Table 1. The mean age of respondents was 48.3 (SD 11.0) years old, and 60.7% were female. Mean full-time employment (FTE) spent on medical education was 39.8% (SD 16.2). Of survey respondents, 85.7% (24/28) were core clerkship directors and 14.3% (4/28) were core clerkship site directors. Most respondents were assistant professors (14/28, 50.0%) or professors (7/28, 25.0%). There were a similar number of respondents from the Midwest (n=9), Northeast (n=8), and South (n=8), with fewer from the West (n=2) and Canada (n=1) (data not shown).

**Table 1:** Demographics of Pediatric Core Clerkship Directors and Medical School Characteristics FTE: full-time employment

	n	Proportion or Mean (Standard Deviation)
Age (years)		48.3 (SD 11.0)
<40	8	28.6%
40-50	9	32.1%
>50	11	39.3%
Gender		
Female	17	60.7%
Male	11	39.3%
Self-Reported Ethnicity/Race		
Asian	5	17.9%
Black	1	3.6%
Hispanic	3	10.7%
Middle Eastern or North African	1	3.6%
Native Hawaiian or Other Pacific Islander	0	0.0%
North American Native	0	0.0%
White	18	64.3%
Academic Rank		
Assistant Professor	14	50.0%
Associate Professor	6	21.4%
Instructor	1	3.6%
Professor	7	25.0%
Current Primary Role as a Medical Educator		
Core Clerkship Director	24	85.7%
Core Clerkship Site Director	4	14.3%
Mean Percent FTE for Medical Education	-	39.8 (SD 16.2)
Region		
Canada	1	3.6%
Midwest	9	32.1%
Northeast	8	28.6%
South	8	28.6%
West	2	7.1%
Mean Class Size		147.2 (SD 59.1)

Respondents reported that medical students spent an average of 8.2% (SD: 10.4) of the core clerkship time participating in the delivery of patient care via telehealth (Table 2). Only 10.7% (3/28) of schools had dedicated instruction in telehealth during the pediatric core clerkship. Of these three, two were in the Northeast, and one was in the Midwest. Instructional methods included lectures, online modules, and simulation - all averaging less than one hour (Table 2). Curricula dedicated the most instructional time to management (38.3%) and by building a history (25.0%). This was followed by communication skills (16.7%) and setting up the encounter (16.7%) (Table 2). Less time was dedicated to physical exam skills (3.3%). Two schools did not conduct assessments, and the other used only formative assessments. No curricula included summative assessments.

**Table 2:** Trends in Telehealth Usage, Content, Assessment, and Barriers During the Pediatric Core Clerkship HIPAA: Health Insurance Portability and Accountability Act

	N	Proportion or Mean	Standard Deviation	Median (IQR)
Estimated average percent of time that medical students participate in the delivery of patient care via telehealth as part of their Pediatric Core Clerkship rotation		8.21%	10.4	5 (1-10)
Does your pediatric core clerkship include dedicated instruction in telehealth?	3	10.7%		
Average time receiving telehealth instruction by instructional strategy among programs with dedicated telehealth instruction (hours)				
Lecture	3	0.3	0.58	-
Simulation	3	0.3	0.58	-
Online Modules	3	0.3	0.58	-
Clinical Care	3	0	0	-
Small groups	3	0	0	-
Other	3	0	0	-
Average percent time dedicated to content areas of telehealth among programs with dedicated telehealth instruction				
Setting up the encounter	3	16.7%	14.43	-
Building a history	3	25.0%	0	-
Communication skills	3	16.7%	14.43	-
Physical examination	3	3.3%	5.77	-
Management	3	38.3%	23.09	-
Other	3	0	0	-
Type of assessment(s) of medical students among programs with dedicated telehealth instruction				
None	2	66.7%	-	-
Formative & summative	0	0%	-	-
Formative only	1	33.3%	-	-
Summative only	0	0%	-	-
Reported barriers that prevent the implementation of a telehealth curriculum as part of the pediatric core clerkship among programs without a dedicated curriculum (N=25)				
Lack of dedicated time in the curriculum	16	64.0%	-	-
Lack of faculty time to teach	11	44.0%	-	-
Lack of faculty expertise	9	36.0%	-	-
Lack of curricular materials	11	44.0%	-	-
Students do not participate in telehealth	10	40.0%	-	-
Lack of financial support	3	12.0%	-	-
Lack of student interest	1	4.0%	-	-
Lack of administrative support	7	33.3%	-	-
Lack of clerkship director interest	2	9.5%	-	-
Other	4	19.0%	-	-
Other reported barriers				
“It has been taught elsewhere in the curriculum, in the orientation to the third year. Also, we keep changing Telehealth platforms.”				
“Issues of HIPAA and family agreement”				
“No video telehealth offered at one of our main sites.”				
“Included in orientation to clinical year.”				

Of schools without telehealth curricula (n=25), the most common barriers to implementing a telehealth curriculum include lack of dedicated time in the existing curriculum (16/25, 64.0%), lack of faculty time to teach (11/25, 44.0%), lack of curricular materials (11/25 44.0%), and lack of faculty expertise (9/25, 36.0%). Over one-third (10/25, 40.0%) reported that no students participated in telehealth activities, a finding more common in the Midwest and the South (data not shown). A smaller percentage of respondents indicated a lack of financial support (3/25, 12.0%) or a lack of student interest (1/25, 4.0%). Clerkship directors also reported telehealth being taught elsewhere in their institution’s medical school curriculum, a lack of stability regarding a telemedicine platform at their institution, non-standardized or lack of availability of telemedicine depending on clinical site, and Health Insurance Portability and Accountability Act (HIPAA) and family agreement issues. 

## Discussion

Telehealth is likely to continue as a critical modality for the clinical care of pediatric patients. While its use will be different than during the height of the pandemic when it was relied upon to sustain the health system through crisis, and the debate continues in regards to its specific advantages in the postpandemic period, there will still be a significant role for telehealth in pediatrics moving forward given its efficacy and convenience [12-14]. Medical students require training to effectively care for patients via telephone and video encounters. In our survey of pediatric clerkship directors, we found that only 10.7% (3/28) of pediatric clerkships offered dedicated instruction in telehealth. Students participated in telemedicine an average of 8.2% of their clinical time on the pediatric core clerkship. Similarly, a survey of internal medicine clerkship directors found only 24.7% of internal medicine core clerkships provided telehealth curriculum or telemedicine experiences as of October 2020 [10]. Though Khullar et al. previously found more than half of medical schools reporting telehealth curricula, it appears the curricula are not integrated into core clerkships such as the pediatrics and internal medicine core clerkships [3].

The use of telehealth has increased in pediatrics and has been shown to be comparable or, in some cases, more effective and efficient than in-person care [14]. A significant number of medical students are interested in learning telehealth, but few are exposed to robust telehealth curricula [15]. Medical students preferred learning telehealth skills early and with an integrated approach throughout their education [16]. A targeted needs assessment in early learners demonstrated the telemedicine physical exam and clinical reasoning skills were areas with the largest gaps in student performance [17]. Faculty time, lack of curricular materials, and lack of faculty expertise were often cited as barriers to implementing a telehealth curriculum. Similarly, other work has found limited curricular time and lack of experienced faculty as the most common barriers to telehealth curricula [10]. However, there have been low-cost, simple telemedicine curricula that minimize faculty time and resources including role play that has been shown to be highly effective in improving student telemedicine skills [18-20].

Limitations

Our study has limitations that affect its generalizability. These include a low response rate, which may reflect responder bias and possibly under-representation of the number of telehealth curricula and could have been a result of survey fatigue during the pandemic [21]. We were also unable to confirm the exact number of medical schools represented by core clerkship directors or site directors who are COMSEP members. However, the data summarizes perspectives directly from curriculum leaders and the geographic distribution of respondents is representative of the regions across the US/Canada, so the data likely represents the prevalence of curricula. We also found a lower, but similar, prevalence of telehealth curricula compared to internal medicine core clerkships [10]. Additionally, our cross-sectional survey may not represent what is currently occurring at institutions. Lastly, many clerkships may have had rapidly changing curricula and clinical experiences across the pandemic, leading to difficulty summarizing telehealth practices in survey responses. 

## Conclusions

Since the COVID-19 pandemic led to changes in the delivery of clinical care, medical schools must take steps towards establishing robust telehealth curricula for medical students, both in the pediatric core clerkship and throughout medical school. Most pediatric core clerkships do not include dedicated telehealth curricula, and the characteristics of existing curricula vary. Considering the rapid adoption of telemedicine, pediatric core clerkships merit additional support and guidance for the training of medical students in telehealth practice. Further research is needed to elucidate the appropriate and effective timing and distribution of telehealth education throughout the medical school experience and evaluate the effectiveness of telehealth curricula and the reliability of current assessments. As telemedicine remains a continued delivery method, UME learners must meet competencies to provide appropriate care. Pediatric core clerkships present a relatively untapped opportunity to provide telehealth instruction and prepare the future workforce to provide care through telehealth. 
